# Role of epidemiology in risk assessment: a case study of five ortho-phthalates

**DOI:** 10.1186/s12940-021-00799-8

**Published:** 2021-11-15

**Authors:** Maricel V. Maffini, Birgit Geueke, Ksenia Groh, Bethanie Carney Almroth, Jane Muncke

**Affiliations:** 1 Independent Consultant, Frederick, MD USA; 2 Food Packaging Forum Foundation, Zurich, Switzerland; 3 Eawag, Swiss Federal Institute of Aquatic Science and Technology, Dübendorf, Switzerland; 4 Department of Biological and Environmental Sciences, University of Gothenburg, Gothenburg, Sweden

**Keywords:** Phthalates, Reference dose, Risk assessment, Epidemiology, Human health

## Abstract

**Background:**

The association between environmental chemical exposures and chronic diseases is of increasing concern. Chemical risk assessment relies heavily on pre-market toxicity testing to identify safe levels of exposure, often known as reference doses (RfD), expected to be protective of human health. Although some RfDs have been reassessed in light of new hazard information, it is not a common practice. Continuous surveillance of animal and human data, both in terms of exposures and associated health outcomes, could provide valuable information to risk assessors and regulators. Using ortho-phthalates as case study, we asked whether RfDs deduced from male reproductive toxicity studies and set by traditional regulatory toxicology approaches sufficiently protect the population for other health outcomes.

**Methods:**

We searched for epidemiological studies on benzyl butyl phthalate (BBP), diisobutyl phthalate (DIBP), dibutyl phthalate (DBP), dicyclohexyl phthalate (DCHP), and bis(2-ethylhexyl) phthalate (DEHP). Data were extracted from studies where any of the five chemicals or their metabolites were measured and showed a statistically significant association with a health outcome; 38 studies met the criteria. We estimated intake for each phthalate from urinary metabolite concentration and compared estimated intake ranges associated with health endpoints to each phthalate’s RfD.

**Result:**

For DBP, DIBP, and BBP, the estimated intake ranges significantly associated with health endpoints were all below their individual RfDs. For DEHP, the intake range included associations at levels both below and above its RfD. For DCHP, no relevant studies could be identified. The significantly affected endpoints revealed by our analysis include metabolic, neurodevelopmental and behavioral disorders, obesity, and changes in hormone levels. Most of these conditions are not routinely evaluated in animal testing employed in regulatory toxicology.

**Conclusion:**

We conclude that for DBP, DIBP, BBP, and DEHP current RfDs estimated based on male reproductive toxicity may not be sufficiently protective of other health effects. Thus, a new approach is needed where post-market exposures, epidemiological and clinical data are systematically reviewed to ensure adequate health protection.

**Supplementary Information:**

The online version contains supplementary material available at 10.1186/s12940-021-00799-8.

## Background

Non-communicable diseases (NCD) are a global burden to public health [1]. Nutritional shortcomings and lifestyle factors have been associated with increased incidence of diabetes and obesity, but current evidence indicates that exposures to environmental chemical contaminants also play a role in the development of NCDs [2]. In the US, cardiovascular diseases and mental health conditions impose the highest economic burden followed by cancer, diabetes, and chronic respiratory diseases [3]. Of particular concern are exposures during gestation and early childhood [4] . A recent review [5] proposed incorporating environmental health risk factors when estimating global burden of disease, including air pollutants, neurotoxicants, endocrine disrupting chemicals, and climate-related factors. To do this successfully, the components of risk assessment such as exposure sources and levels, as well as data about chemical effects and associated health outcomes, are required [6].

One source of chemical exposure is plastic. With a global production of almost 360 million metric tons in 2018 [7], manufacturing, use, and disposal of plastic materials pose major safety concerns. Leachate from landfills, migration from consumer products (e.g., food packaging, toys, flooring, textiles), and air pollution from burning plastic materials are just some of the sources of chemical contamination affecting humans and the environment [8–10] . Because information on chemicals present in plastics is difficult to obtain and their hazards often remain unknown, Groh and colleagues [11] published a comprehensive database with more than 900 chemicals likely associated with plastic packaging as part of the Hazardous Chemicals in Plastic Packaging (HCPP) project. The authors also ranked the chemicals based on hazards to human and environmental health according to the United Nations’ Globally Harmonized System of Classification and Labelling of Chemicals [12] The 63 chemicals that ranked highest for human health concerns underwent a tiered prioritization [13] based on biomonitoring data, endocrine disrupting properties, and their regulatory status under the European Chemicals Regulation REACH. This prioritization approach identified five ortho-phthalates (referred to as phthalates in this article) for which the risk to human health was considered the highest: benzyl butyl phthalate (BBP, CAS 85-68-7); dibutyl phthalate (DBP, CAS 84-74-2); diisobutyl phthalate (DIBP, CAS 84-69-5); bis(2-ethylhexyl) phthalate (DEHP, CAS 117-81-7); dicyclohexyl phthalate (DCHP, CAS 84-61-7).

Phthalates are highly abundant plastic additives used primarily as plasticizers to soften materials and make them flexible [14]. Human biomonitoring shows widespread exposure to phthalates [15] from diverse sources including food which could be contaminated from its packaging as well as other food contact materials such as conveyor belts and tubing used in food processing [16–20]. Personal care products and building materials also contribute to human exposure to phthalates [21, 22].

Several regulatory authorities have assessed the toxicity of BBP, DBP, DIBP, DEHP, and DCHP [23–25] and established the amount of each chemical above which the risk to human health increases. Regulatory agencies give different names to these so-called ‘safe’ levels including derived no-effect level (DNEL) used by the European Chemical Agency (ECHA) [26], acceptable daily intake (ADI) used by International Programme on Chemical Safety [27], total dietary intake (TDI) used by the European Food Safety Authority (EFSA) [28] and reference dose (RfD) used by the U.S. Environmental Protection Agency (EPA) [29]. Although the nomenclature is different, the meaning is similar, namely, exposures above established amounts of chemicals are not safe. For simplicity, we use the term RfD throughout the article.

The established RfDs for the five phthalates are the result of risk assessments of mostly animal studies showing adverse effects on male reproductive development due to the anti-androgenic properties of these chemicals. These risk assessments’ results have led to the restriction of some uses of these phthalates. In 2008, the Congress of the United States banned the use of DEHP, BBP and DBP in children’s toys and childcare articles [30] and in 2017, the Consumer Protection Safety Commission increased the list of prohibited phthalates to eight [23]. Similarly, the European Union has also listed DEHP and DBP in its authorization list under REACH and more than a dozen phthalates are included in the candidate list for authorization [31]. The observed decline in human exposure to restricted phthalates in industrialized countries over the years [15, 32, 33] have been attributed to these regulatory measures. Notably there are yet no major restrictions to uses in food contact materials (e.g., packaging, processing equipment), pharmaceuticals, and medical devices.

Epidemiological data published in the last 15 years indicate that in some cases exposure to phthalates is still a cause of concern to human health. For example, recent publications by the U.S. Environmental Protection Agency (EPA) show a strong association between exposure to low concentrations of DEHP, DBP, and DIBP and increased risk of diabetes [34], and between exposure to DEHP and DBP and male reproductive effects such as reduced semen quality and testosterone levels [35]. Several small- and large-scale human studies have also shown phthalates to associate in a dose-dependent manner with negative effects on neurodevelopment [36, 37], metabolic function [38] and female reproduction [39]. Therefore, we aimed to investigate whether regulatory safe levels of phthalates are protective of the public for other relevant health outcomes in addition to male reproductive development. We conducted a targeted literature search of human studies showing association between any of the five phthalates, BBP, DBP, DIBP, DEHP, and DCHP and health effects. Furthermore, we back-estimated daily intake for each phthalate that showed a statistically significant association with health effects, and compared these estimated intake values to the individual RfD.

## Methods

### Targeted literature search

We searched the Public Library of Medicine for human studies on phthalates published between 2003 and 2019. Search terms included compounds’ full name, abbreviation, and chemical abstracts service (CAS) numbers in combination with human exposure, epidemiological studies and metabolites among others. See Supplemental Materials for additional information. This targeted search aimed at obtaining information on the five phthalates including concentration of parent phthalates or metabolites in any bodily fluid, description of measured endpoint, and statistical significance of the association between health endpoint and concentration measured. When a study met these criteria, we extracted the following data: 1) population sampled and population in which the endpoints were measured (e.g., men; pregnant women/children; children, etc.); age; gestational age where appropriate; 2) metabolite or parent compound concentration as percentile, geometric mean or other available concentration measure; 3) concentration at which metabolite(s) or parent compound had a statistically significant correlation with an endpoint; 4) statistically significant endpoint and outcome (e.g., increase/decrease; positive/negative association). We used the studies that met the criteria described above to perform the analysis and controlled for quality, specifically, whether the studies included controls for covariates and confounders such as race, maternal/paternal age, child’s sex, IQ, socioeconomic status, smoking, physical activity, caloric intake, etc.; however, we did not control for potential bias.

### Intake estimation from urinary concentration

From the studies that met the inclusion criteria, we identified the lowest phthalate metabolite concentration that was associated with a statistically significant endpoint. Concentration data were expressed in various ways including geometric means of a population, percentiles, and average of urine collections per individual visits. We established the following assumptions: 1) the 25th percentile concentration was considered equivalent to a no-observed-adverse-effect level when concentrations were expressed as quartiles, meaning that only concentrations at or greater than the 25th percentile were included; 2) unless specified in the studies, logistic regressions were considered linear.

For each phthalate, we estimated intake using urinary concentration of its metabolite(s), daily urine volume, body weight (bw), and creatinine correction values for the different populations assessed [40, 41]. In the case of DEHP, we considered the individual excretion of its four metabolites over time and expressed it as percent of the parent phthalate’s intake as described previously [42, 43]. We used the following mean percentage excretion values for DEHP metabolites: 6% for monoethylhexyl phthalate (MEHP), 11% for mono-(2-ethyl-5-oxohexyl phthalate (5oxo MEHP), 15% for mono(2-ethyl-5-hydroxyhexyl) phthalate (5OH MEHP) and 14% for mono-(2-ethyl-5-carboxypentyl) phthalate (5cx MEHP). For DBP, DIBP and BBP, we followed the European Chemical Agency (ECHA) assumption of 100% elimination of the parent compound as phthalate monoesters [25]. We used the following formula:

Intake (μg/ bw (kg)/d) = Metabolite concentration (μg/L) x (Vol (L)/day) x (1/bw (kg)) x (1/% elimination)

In cases when creatinine correction was needed, concentration of urinary metabolite in microgram per gram (μg/g) creatinine was multiplied by the urinary concentration of creatinine in gram per liter (g/L). The Supplemental Materials include an example of the intake calculations and the assumptions made for each population (children, pregnant women, non-pregnant women, men) regarding body weight, daily urine volume, and creatinine excretion.

### Regulation of priority phthalates

The uses of and exposure to phthalates are regulated in the European Union and the United States [23–25, 44, 45]. We chose the regulatory limits set by ECHA and the US Consumer Protection Safety Commission (CPSC) to compare against the estimated intakes associated with health endpoints because these safe levels have been reaffirmed or established in the last 5 years using current scientific evidence. In addition, both assessments target products that are commonly used by children, a susceptible population as highlighted by government regulatory agencies [23, 25, 46]. Regulatory RfDs are commonly expressed as the amount of chemical a person is safe to consume per kilogram of body weight per day, over their expected lifetime. Table 1 summarizes the RfD for BBP, DBP, DIBP and DEHP and the health endpoint selected by ECHA to establish each reference dose. Because ECHA did not establish an RfD for DCHP, we used a regulatory limit set by the US CPSC, i.e., less than 0.1% DCHP per weight of the final product for children’s toys and articles [23]. This assessment was also based on DCHP’s anti-androgenic effects (i.e., reduced anogenital distance) observed in male rodents [54].

**Table 1:** Comparison between reference doses (RfDs) set by European Chemicals Agency (ECHA), including their corresponding endpoint of concern in animal studies, and range of phthalate estimated intakes that were reported to be significantly associated with endpoints of concern in humans

Phthalate	*Derived from animal studies*		*Based on human studies*			
	RfD ^**a**^	Endpoint and effect of concern	Lowest estimated intake ^**a**^	Significant endpoint	Highest estimated intake ^**a**^	Significant endpoint
DEHP	35	Testicular germ cell depletion and reduced testes weight	0.03	Decreased number of ovarian antral follicles in women [39]	242.5	Decreased semen quality and concentration in men [47]
DBP	6.7	Reduced spermatocyte development at postnatal day 21, and mammary gland changes in adult male offspring	0.19	Decreased sperm motility and semen concentration in men [48]	2.86	Decrease thyroid hormone T4 and freeT4 in women [49]
BBP	500	Reduced anogenital distance and several other endpoints from various studies	0.06	Increased steroid hormone binding globulin in children [50]	0.58	Increased body mass index and waist circumference in men and women [51]
DIBP	8.3	Overall potency of DIBP similar to DBP; possible potency difference of 25% between DIBP and DBP	0.08	Decreased masculine play behavior in boys [52]	0.51	Increased occurrence of eczema in children [53]

^a^ Units are in microgram per kilogram of body weight per day

*DEHP* diethylhexyl phthalate; *DBP* dibutyl phthalate; *BBP* butylbenzyl phthalate; *DIBP* diisobutyl phthalate;

## Results

We identified 38 out of 64 publications that met our selection criteria (Table 2). The studies included longitudinal and cross-sectional studies; small cohorts (e.g., patients at fertility clinics; under-represented urban populations) and nationally representative cohorts such as the National Health and Nutrition Examination Survey (NHANES) of the U.S. Center for Disease Control and Prevention; and prenatal exposure studies where phthalates were measured in the mothers but the health outcomes were assessed in their children months or years after birth. Supplemental Materials Table S1 lists the 26 publications that did not meet our criteria and therefore were not included in this case study.

**Table 2:** Summary of 38 studies that met the criteria for data extraction and estimated intake associated with statistically significant endpoints, grouped by population sampled

Population sampled	Significant endpoint	Significant outcome	Parent phthalate	Estimated phthalate intake ^**a**^	Lower concentration statistically significant	Reference
Women	Number of ovarian antral follicles	Decreased	DEHP	0.03–0.25^b^	1.63 μg/L-13.5 μg/L	[39]
		Decreased	DIBP	0.13–0.19^c^	10.21 μg/L	
		Decreased	DBP	0.24–0.42	12.79 μg/L	
	Glucose levels	Decreased	DIBP	0.20	10.7 μg/L	[38]
	Thyroid hormone T4	Decreased	DBP	0.20	9.6 μg/g CRE	[55]
	Free thyroid hormone T4	Decreased	DEHP	0.53–1.80	1.69 μg/L-13.4 μg/L	[56]
	Thyroid hormone T4 and free T4	Decreased	DBP	2.86	9.6 μg/g CRE	[49]
	Total number of oocytes, fertilized oocytes, mature oocytes, top quality embryos	Decreased	DEHP	0.23–1.39	0.02 uM-0.12 uM	[57]
	Total number of fertilized oocytes, mature oocytes, top quality embryos	Decreased	DBP	0.24	12.7 uM	
	Trophoblast differentiation genes	Decreased	DiBP	0.27	14.2 μg/L	[58]
		Decreased	DBP	0.71	38 μg/L	
		Decreased	DEHP	4.21	221.2 μg/L	
	Body mass index and waist circumference	Increased	DBP	0.30	12.26 μg/g	[59]
	Body mass index	Increased	DEHP	0.56	1.49 μg/g CRE	
	Homeostatic Model Assessment of Insulin Resistance (HOMA-IR)	Increased	DEHP	1.56	12.51 μg/L	[60]
	Serum inhibin	Decreased	DEHP	2.04	5.44 μg/g CRE	[61]
	Gestational age	Shorter	DEHP	2.46	18.36 μg/L	[62]
		Longer	DEHP	2.56–4.20	1.1 μg/L-5.1 μg/L	[63]
	C-section	Increased likelihood	DEHP	2.56–4.20	1.1 μg/L-5.1 μg/L	[63]
Mothers (3 T)	Serum steroid hormone binding globulin	Increased	BBP	0.06	3.16 μg/L	[50]
		Increased	DBP	0.63	33.4 μg/L	
	Serum dehydroepiandrosterone sulfate	Decreased	DBP	0.63	33.4 μg/L	
	Social problems	Increased	BBP	0.07–0.23	3.2 μg/g	[64]
	Delinquent and externalizing behavior	Increased	DEHP	2.36	0.17 µmol/g CRE (sum DEHP)	
	Internalizing and externalizing problems	Increased	DEHP	6.27	16.7 μg/g CRE	
	Motor development	Delayed	DIBP	0.17	9.3 μg/L	[65]
	Psychomotor development index	Decreased	DIBP	0.17	9.3 μg/L	
	Clinically withdrawn behavior and internalizing behavior	Increased	BBP	0.36	19 μg/L	
	Psychomotor and mental development index	Decreased	DBP	0.71	38 μg/L	
	Clinically withdrawn behavior	Increased	DBP	0.71	38 μg/L	
	Full scale IQ, perceptual reasoning, processing speed, verbal comprehension and working memory	Decreased	DIBP	0.36	19 μg/L	[36]
		Decreased	DBP	1.5	79.8 μg/L	
	Perceptual reasoning	Decreased	BBP	0.56	30 μg/L	
	Mental and psychomotor development indices	Decreased in boys	DBP	0.38	16.9 μg/g CRE	[66]
	Psychomotor development index	Decreased in boys	DEHP	2.7	13.2 μg/g CRE	
	Body mass index z-score	Decreased in girls	DEHP	1.77	0.128µM	[67]
Mothers (2 T)	Masculine play behavior	Decrease	DIBP	0.08	4 μg/L	[52]
		Decrease	DEHP	0.38–0.88	1.4–4.7 μg/L	
	Internalizing behavior	Increased	BBP	0.26	12.8 μg/L	[68]
		Increased	DBP	0.68	33.1 μg/L	
	Emotional symptom score and relationship problems	Increased	DBP	0.68	33.1 μg/L	
	Eczema	Increased	DIBP	0.51	25 μg/L	[53]
	Attention deficit hyperactivity disorder	Increased	DEHP	2.66	0.21 µM	[69]
Mothers (1 T)	Anogenital distance	Shorter	DEHP	0.66–0.80	2–6.1 μg/L	[70]
Children (4–9 yo)	Thyroid hormone T3 and free T3	Decreased in girls	BBP	0.10	3.3 μg/L	[71]
		Decreased in girls	DBP	1.97	63 μg/L	
		Decreased in boys	DBP	2.34	75 μg/L	
Children (12 yo)	Height standard deviation	Decreased in obese pubertal children	DEHP	0.18	0.6 μg/g CRE	[72]
	Insulin sensitive index	Increased in obese pre-pubertal children	DEHP	0.19	0.27 μg/g CRE	
	Waist circumference	Decreased in obese pre-pubertal children	DEHP	0.19–0.35	0.27–1.24 μg/g CRE	
	Puberty	Delayed in obese pre-pubertal children	DEHP	0.19–0.35	0.27–1.24 μg/g CRE	
	Waist to hip ratio	Increased in obese pre-pubertal children	DEHP	0.23	0.76 μg/g CRE	
Children (8–14 yo)	Estrogen, testosterone and free testosterone	Decreased	DIBP	0.18	7.02 μg/L	[50]
	Free testosterone	Decreased	DEHP	1.52	3.48 μg/L	
	Serum steroid hormone binding globulin	Increased	DEHP	1.52–5.09	3.48–29 μg/L	
Children (8 yo)	Puberty	Delayed in girls	BBP	0.19	6.2 μg/L	[73]
		Delayed in girls	DBP	1.56	50 μg/L	
		Delayed in girls	DEHP	4.63	74 μg/L	
Children (8–10 yo)	Obesity	Increased in boys	DBP	0.95	30.4 μg/L	[74]
		Decreased in girls	DEHP	1.72–4.31	3.3–32 μg/L	
Children (6–19 yo)	Albumin/creatinine ratio	Increased	DEHP	1.60	0.1 µM	[75]
	Systolic blood pressure	Increased	DEHP	3.20	0.166 µM	[76]
Children (12–19 yo)	Thyroid hormone T3	Increased	DEHP	2.2–2.89	5.76–10.3 μg/g CRE	[77]
	Homeostatic Model Assessment of Insulin Resistance (HOMA-IR)	Increased	DEHP	2.76	0.17 µM	[78]
Men	Sperm motility and concentration	Decreased	DBP	0.19	10.6 μg/L	[48]
		Decreased	DBP	0.38		[79]
	Sperm motility	Decreased	BBP	0.25	13.4 μg/L	[79]
	Semen quality	Decreased	DEHP	242.55	20.16 μg/L	[47]
	Testosterone, free testosterone, free androgen index	Decreased	DEHP	0.40–2.13	1.3–15.9 μg/L	[80]
	Serum steroid hormone binding globulin	Increased	DEHP	0.41	1.3 μg/L	
	Testosterone, estrogen, free androgen index	Decreased	DEHP	0.99	3.18 μg/L	[81]
	Testosterone/estrogen ratio	Increased	DEHP	0.99	3.18 μg/L	
	Thyroid hormone T3 and free thyroid hormone T4	Decreased	DEHP	0.99	3.18 μg/L	[82]
	Thyroid stimulating hormone	Decreased	DEHP	242.55	21 µM	[47]
Men and women	Body mass index and waist circumference	Increased	BBP	0.58	30.9 μg/L	[51]
		Decreased	DEHP	13.75	44 μg/L	
	Body mass index	Increased	DBP	0.72	38.4 μg/L	
	Homeostatic Model Assessment of Insulin Resistance (HOMA-IR)	Increased	DEHP	3.16	18.51 μg/L	
	Thyroid hormone T4	Decreased	DEHP	1.48–1.97	5.43–9.84 μg/g CRE	[77]
	Thyroid stimulating hormone	Increased	DEHP	1.97	9.84 μg/g CRE	

^a^ In microgram per kilogram of body weight per day. See Supplemental Materials

^b^ For DEHP, a range is given when more than one metabolite was statistically significant for an endpoint

^c^ A range is given when statistical significance was observed at one or more tertile/quartiles

^d^ *Q* quartile

Abbreviations: *CRE* creatinine; *DEHP* diethylhexyl phthalate; *DBP* dibutyl phthalate; *BBP* butylbenzyl phthalate; *DIBP* diisobutyl phthalate; *IQ* intelligence quotient; *1 T, 2 T, 3 T* first, second and third trimester; *yo* year-old

All 38 studies reported phthalate metabolites measured in urine. DEHP was the phthalate most frequently assessed. There were 12 studies on mother-child pairs evaluating prenatal exposure effects, 12 women-only studies, six men-only studies, eight children studies evaluating postnatal exposure effects, and two studies including both men and women. A few studies included more than one population (e.g., children and adults) and only one study was a prospective mother-child study. It is worth noting that none of the studies included evaluation of DCHP, neither as a parent compound nor its metabolite. The lack of epidemiological studies on DCHP is likely due to the fact that the urinary concentration of DCHP metabolite has been found to be consistently below the limit of detection at the 75th percentile in the NHANES 1999–2010 period [83] and, when measured, the frequency of detection has been low (e.g., less than 10% of the population tested) [33, 83].

Table 1 lists the range of exposure for each phthalate and their association with significant endpoints. All phthalates measured in urine as metabolites of DEHP, DBP, BBP and DIBP showed significant associations with reproductive (male and female), neurodevelopmental, behavioral, hormonal, and metabolic endpoints at estimated intake values well below their respective RfDs.

**Fig. 1:**
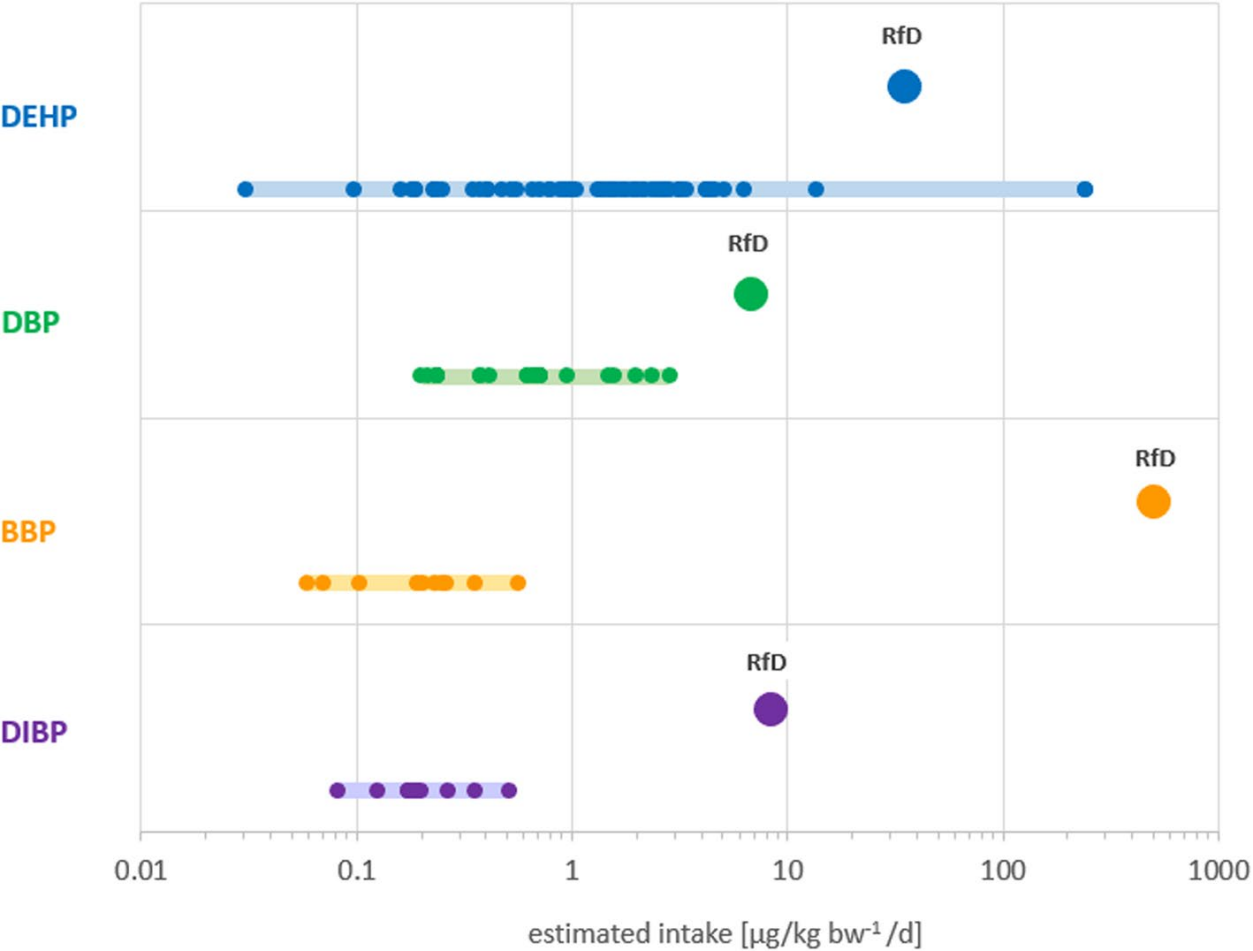
Schematic representation of the range of estimated intake for individual phthalates (solid light-colored bars) associated with statistically significant endpoints (small circles) in relation to their respective reference doses (RfD; large circles). Each small circle corresponds to an endpoint significantly associated with an estimated intake. The lowest metabolite concentrations measured in urine that were found to be associated with statistically significant endpoints were 0.03, 0.19, 0.06 and 0.08 μg/L for DEHP, DBP, BBP and DIBP, respectively. See Supplemental Table S2 for additional data. DEHP: diethylhexyl phthalate; DBP: dibutyl phthalate; BBP: butylbenzyl phthalate; DIBP: diisobutyl phthalate

Figure 1 shows the estimated intake distribution per phthalate compared to the respective RfD. DEHP had the widest range of estimated intakes associated with statistically significant endpoints: 0.03–242.5 μg/kg-bw/d (Table 1, Fig. 1). The highest estimate was almost seven times greater than the RfD (35 μg/kg-bw/d) which is an indication that some individuals could already be exposed to unsafe levels of the chemical as judged by the current regulatory limits. As shown in Table 1, the highest DEHP intake was associated with decreased semen quality [47]. On the lower end, DEHP was associated with significantly lower number of ovarian antral follicles (a measure of remaining oocytes supply) [39] at an estimated intake three-orders of magnitude lower than the RfD (0.03 and 35 μg/kg-bw/d, respectively).

For DBP, DIBP and BBP, the ranges of intake associated with statistically significant endpoints were all below their respective RfDs. (Fig. 1). The lowest estimated intake for DBP (0.19 μg/kg-bw/d) was associated with decreased sperm motility and semen concentration [48] while the highest intake (2.86 μg/kg-bw/d) was associated with decreased concentration of total thyroid hormone thyroxine (T4) and free T4 (fT4) in women [49]. The lowest DIBP intake measured in pregnant women (0.08 μg/kg-bw/d) was associated with decrease in masculine play behavior in boys [52] and the highest intake (0.51 μg/kg-bw/d) also measured in pregnant women, was significantly associated with increased occurrence of eczema in children [53]. The range of estimated intake for BBP associated with significant endpoints showed the greatest difference with the RfD. The lowest intake of 0.06 μg/kg-bw/d was associated with increased levels of steroid hormone binding globulin (SHBG) in children [50]. SHBG is a protein that transports estrogen and testosterone in the blood and regulates their access to tissues [84]. The highest estimated intake for BBP (0.6 μg/kg-bw/d) was associated with increased body mass index and waist circumference in men and women [51]. These intakes are eight-thousand to five-thousand times lower than BBP’s RfD of 500 μg/kg-bw/d.

The four phthalates for which we found data are known to affect male reproductive development due to their anti-androgenic properties which are the basis of their regulation. However, other systems are also affected at exposure levels similar to those associated with anti-androgenicity as seen in Table 2. Our analysis shows the 10 lowest estimated intakes were significantly associated with endpoints measured in women and children. Many of these endpoints relate to endocrine function and neurobehavioral development in children as well as female reproductive system (Table 3).

**Table 3:** Ten lowest estimated intake and significant endpoints by population

Phthalate	Endpoint statistically significant	Effect	Population Tested	Estimated intake ^**a**^	Reference
DEHP	Number of ovarian antral follicles	Decreased	Women	0.03–0.16 ^b^	[39]
BBP	Serum steroid hormone binding globulin in children	Increased	Mothers 3 T	0.06	[50]
BBP	Social problems in children	Increased	Mothers 3 T	0.07	[64]
DIBP	Masculine play behavior in boys	Decreased	Mothers 2 T	0.08	[52]
BBP	Thyroid hormone T3	Decreased	Children (4–9 yo)	0.10	[71]
DIBP	Number of ovarian antral follicles	Decreased	Women	0.13	[39]
DIBP	Motor and psychomotor development in children	Delayed and Decreased	Mothers 3 T	0.17	[66]
DEHP	Height standard deviation	Decreased	Children (12 yo)	0.18	[72]
DIBP	Estrogen, testosterone and free testosterone	Decreased	Children (8–14 yo)	0.18	[50, 53]
DEHP	Insulin sensitivity	Increased	Children (12 yo)	0.19	[72]

^a^ In microgram per kilogram of body weight per day

^b^ Range of intake estimated based on urinary concentration of monoethylhexyl phthalate (MEHP), mono(2-ethyl-5-oxohexyl) phthalate (MEOHP) and Mono(2-ethyl-5-hydroxyhexyl) phthalate (MEHHP)

Abbreviations: *DEHP* diethylhexyl phthalate; *DBP* dibutyl phthalate; *BBP* butylbenzyl phthalate; *DIBP* diisobutyl phthalate; *1 T, 2 T, 3 T* first, second and third trimester; *yo* year-old

Prenatal exposures to DEHP, DBP, BBP and DIBP were significantly associated with a diverse set of negative outcomes in the neurological system, and all endpoints were associated with intakes well below the RfD for each phthalate. Supplemental Table S2 shows that children born to mothers exposed to phthalates during pregnancy display delayed psychomotor and mental development [65, 66]; decreased intellectual, memory and executive function development [36]; and behavioral changes associated with both delinquency and externalization [64] as well as withdrawn personalities and internalization of problems [65, 68]. Increased odds of attention deficit hyperactivity disorder [69] and decreased masculine behavior in boys [52] were also observed.

We identified three major systems associated with metabolic function that were affected by phthalates: thyroid, pancreas, and fat tissue (Supplemental Table S3). DEHP, DBP and BBP were associated with decreased levels of triiodothyronine (T3) in men and children as young as 4 years of age. DEHP was also associated with decreased levels of free T4 in women [56] and men [82] and decreased thyroid stimulating hormone (TSH) in men [47].

DIBP and DEHP intakes were positively associated with insulin resistance in children [72, 78] and men and women [60, 77]. The effect of DEHP on fat tissue was more diverse. For instance, in adults, body mass index (BMI) was negatively associated with DEHP levels in men and women [59], while Hatch et al. [51] reported a positive correlation in women). Maternal DEHP levels were inversely associated with their daughters’ BMI at a young age (4–7 years) [67] and Zang and colleagues also observed a negative association between DEHP levels and obesity in 8–10-year-old girls [74]. DBP and BBP showed a positive correlation with obesity in boys [74], BMI and waist circumference in women and men [51].

All the estimated intakes were below their respective RfDs, except for the reduction in TSH level in men that was associated with the highest DEHP intake of 242.55 μg/kg-bw/d [47].

Both, the male and female reproductive systems and their associated hormones, were negatively affected by the four phthalates (Supplemental Table S4). DEHP, DBP and DIBP intakes were associated with reduced number of antral follicles in women [39] and DEHP, DBP and BBP with delayed puberty in girls [73]. DEHP and DBP were associated with decreased number of fertilized eggs and total oocytes, and lower quality of oocytes [57]. DEHP and DIBP showed a negative association with trophoblast differentiation genes [58]. DEHP was also associated with decreased levels of inhibin [61], a critical hormone in reproductive functions [85], and showed inconsistent association with gestational length [62, 63].

In adult men, DEHP, DBP and BBP all had a negative association with semen quality including concentration and sperm motility [47, 48, 79]. DEHP was associated with decreased total and free testosterone and estradiol, as well as increased levels of SHBG [80]. DEHP also had a positive association with testosterone/estradiol ratio [81]. In boys gestationally exposed to known levels of phthalates, DEHP and DIBP were negatively associated with free and total testosterone and estradiol [50]. DEHP, DBP and BBP were associated with increased SHBG. DBP was associated with decreased levels of dehydroepiandrosterone [50]. Finally, DEHP was also associated with reduced anogenital distance in boys [70].

## Discussion

This case study shows that low dose exposures to BBP, DBP, DIDP and DEHP are associated with health endpoints in organs and systems not usually assessed in regulatory toxicology studies. These endpoints differ markedly from the well-studied effects of phthalates on male reproductive development. Furthermore, there are significant physiological effects (i.e., early biological perturbations that may lead to overt effects) and disorders that may require clinical interventions later in life associated with estimated intake levels lower than the current RfD. We also observed that some individuals appear to be exposed to levels of DEHP higher than its RfD. This may be the case if there are yet to be identified exposure routes and sources, or if the metabolism or excretion of DEHP is altered. Overall, these data, although with limitations, show weaknesses in a chemical regulation framework that is in need of improvement.

Some of the limitations are, first, this study is similar to a mapping of evidence; it is not a systematic review that must follow stricter protocols and methods. Second, our approach aimed to capture as many publications as possible. However, although we used broad search terms, we may still have missed relevant publications. Third, we trust the integrity and quality of the journal peer-reviewed conducted for each of the studies we included. However, we understand the peer review process is not perfect. An example of this less-than-perfect process is the lack of clarity or data that prevented us to include an additional 26 human studies as shown in Table S1. Importantly, only six studies were excluded because of the lack of statistical significance, hence, the body of evidence is consistent with the associations. Fourth, in some cases, data interpretation had to be based on information that was available. Although we contacted authors from some of the studies that did not meet our criteria to obtain additional data, only a few responded to our request and were willing to share additional data. Fifth, the number of subjects in the studies varied from less than 100 to thousands of people; although the population size as such could be a limitation, strong and weak statistical significance was observed in all cases. As all but one study was cross-sectional, we are mindful about implying that they show causality. Lastly, some assumptions made in our calculations may have been outdated. For example, the EPA handbook on exposure is from 2011. Although it is our understanding that the agency and others continue to use this handbook in their analysis, we cannot rule out that parameters such as *body weight by age range* may have changed in the last decade and could have affected our estimates.

Overall, the case study we present here specifically aimed to use strong human data to perform a first examination of a hypothesis, namely that the current animal-based testing methods to estimate “safe” exposure levels of chemicals could be significantly underestimating actual human health risk if epidemiological data are not considered. Following the initial confirmatory findings presented here, this hypothesis will serve as a basis to guide further testing and more detailed assessments in a follow-up work.

The protection of public health from detrimental effects of environmental chemical exposures should ideally incorporate the expertise from two sides: the risk assessors and the healthcare community, including epidemiologists. On the one hand, risk assessment relies on evaluating exposure to a chemical and using animal models to identify which organ(s) would be affected, in order to find a dose that would cause no harm. On the other hand, the medical community is confronted with a wide range of health outcomes in the human population—from acute to chronic and from subtle to clinically defined—and tries to identify what caused them, whether environmental chemical exposure or otherwise, in order to support prevention. But there is a disconnect between these bookends of environmental health which hinders effective protection of the public from chemical exposures. In 2017, the US National Academy of Sciences [86] recommended that for evaluating evidence of low dose effects, regulators should surveil for signals indicating an adverse outcome in a human population or evidence that a particular low dose effect may not be detectable with traditional toxicity testing. The authors stated that one way to seek out information is by conducting regular surveys of the scientific literature. Our limited case study of five phthalates shows that many of the health effects observed to occur in humans at very low exposure levels are not traditionally evaluated in animal toxicology testing. Metabolic, neurodevelopmental and behavioral disorders, obesity, levels of hormones and transport proteins are just a few examples of endpoints not commonly included in toxicity testing guidelines despite their relevance to human health. It is also important to point out that traditional toxicology studies only infrequently evaluate a dose-effect relationship using chemical levels relevant to human exposures occurring at different life-stages. Rather, assumptions of safe levels are commonly made based on adult non-pregnant animal data. These omissions thus result in significant gaps in chemicals regulation that may put human health at risk [87].

The current chemical risk assessment approach to establish an RfD used by most regulatory agencies around the world combines a dose that did not cause an adverse effect in animal studies using high exposure doses and safety factors (also known as uncertainty factors) to account for incomplete data and variability between and within species. Although not routinely, regulatory ‘safe’ levels have been reviewed. For example, ECHA lowered the derived no effect level for DIBP from 420 to 8.3 μg/kg bw/d in 2016 [25]; similarly, the European Food Safety Authority (EFSA) lowered the tolerable daily intake of bisphenol A from 50 to 4 μg/kg bw/d in 2015 [88]. In both cases, new scientific information was available at the time the agencies were responding to requests for reassessment of those chemicals. However, we would argue that, in addition to specific requests made to regulatory agencies, a more systematic reevaluation of RfDs could be incorporated into the risk assessment and management processes. For example, a post-market RfD reassessment could be triggered by 1) human studies showing associations between exposure and endpoints previously not measured; 2) information on reported uses or biomonitoring indicating increased exposures due to chemical production volumes or reduced exposure due to abandoned uses; or 3) new hazard information. Lastly, this information surveillance should not be the exclusive responsibility of the regulatory agencies; rather, companies with approved chemical uses should submit new available information that could potentially raise questions about the safety of their product and agencies should establish a mechanism to enforce this requirement.

Both, scientific information and market behavior, are dynamic. Advances in science and technology allow scientists to develop new methods to measure chemicals in humans and gain new knowledge and understanding of chemicals’ interactions with physiological systems at different life stages. To account for these developments, epidemiological and clinical studies together with chemical biomonitoring data should be evaluated at regular intervals as recommended by the NAS [86] in order to check whether an RfD review is warranted to better protect public health. We are cognizant that this approach, although promising, is not without shortcomings. For instance, biomonitoring data alone cannot account for all sources of exposure. For chemicals like phthalates, with many sources ranging from the diet to personal care products and house dust, it may be challenging to design mitigating strategies to reduce the most significant sources of exposure. However, well designed surveys and a better understanding of materials’ composition may help identifying the major exposure sources for various populations as it was described by Lioy and colleagues [89].

As implied earlier, the RfD represents a concept of ‘safety’, a bright line between ‘no risk’ or ‘safe’ when the exposure estimate is below the established number and ‘risk’ or ‘unsafe’ when the value is greater than the RfD. In reality, it is far more complicated, namely, chemical hazard information and populations’ background exposures from multiple chemicals, health conditions and life-stages change with time. In its 2009 Science and Decisions report [90], the NAS recognized this complexity and recommended a progression away from the current concept of ‘safety’ and towards dose-response methods that quantify risk at doses used in animal experiments as well as lower doses representing human exposures. As much as two-thirds of the human population suffers from chronic diseases that cannot be explained by genetic causes alone [91] and it is becoming increasingly apparent that life-long chemical exposures can contribute to this burden [5]. Yet, for the great majority, chemicals are not evaluated for their contribution to common chronic ailments in the human population [92, 93]. As a consequence, the current work on toxicology and epidemiology is inundated with disconnected data that misses the bigger picture: better protection of the entire human population’s health. Perhaps it is time to reconsider the status quo to ensure adequate population health protection. Issues to be interrogated may include, among others, strategies for proper assessment of the risk of developmental exposures; use of early biomarkers of health effects; integration of evidence from different data streams including predictive modeling, in vitro, animals and humans; development of new and redesign of old testing protocols; optimization of in vitro testing to minimize the use of laboratory animals; design of protocols to more efficiently monitor human exposures.

## Conclusions

Phthalates have been used in many products for many decades. There are substantial animal and human data available which allowed us to use these substances in case studies such as this one. However, a similar question could be raised for many other chemicals with a growing body of human biomonitoring data and evidence of human health effects [94].

To set the course for a better, more efficient and health protective risk assessment of chemicals, a dialogue should be established between risk assessors, the medical community, and academic researchers. Until a profound modernization of the risk assessment and management of chemicals occurs, human studies should be taken into account to identify whether the health risk of chemicals already in the marketplace, such as phthalates, should be reassessed.

## Supplementary Information

**Additional file 1.**

## Abbreviations

μg: Microgram
1 T: First trimester
2 T: Second trimester
3 T: Third trimester
ADI: Acceptable daily intake
BBP: Benzyl butyl phthalate
BMI: Body mass index
Bw: Body weight
CAS: Chemical abstract service
CPSC: Consumer products safety commission
D: Day
DBP: Dibutyl phthalate
DCHP: Dicyclohexyl phthalate
DEHP: Bis(2-ethylhexyl) phthalate
DIBP: Diisobutyl phthalate
DNEL: Derived no-effect level
ECHA: European chemical agency
EFSA: European food safety authority
EPA: Environmental protection agency
HOMA-IR: Homeostatic model assessment of insulin resistance
IQ: Intelligence quotient
Kg: Kilogram
L: Liter
NCD: Non-communicable disease
NHANES: National health and nutrition examination survey
REACH: Registration, evaluation, authorization and restriction of chemicals
RfD: Reference dose
SHBG: Steroid hormone binding globulin
T3: Triiodothyronine
T4: Thyroxine
TDI: Tolerable daily intake
TSH: Thyroid stimulating hormone
US: United States
Yo: Year old
