# Supplementary material for: Role of epidemiology in risk assessment: a case study of five ortho-phthalates
Source: Environ Health. 2021 Nov 15;20:114. doi: 10.1186/s12940-021-00799-8 (PMC8591894; doi:10.1186/s12940-021-00799-8)
Supplement: Supplementary file 1 — Additional file 1. [file 12940_2021_799_MOESM1_ESM.docx]

Title: Role of Epidemiology in Risk Assessment: A Case Study of Five Ortho-phthalates

Authors: Maricel V. Maffini, Birgit Geueke, Ksenia Groh, Bethanie Carney Almroth, Jane Muncke

Supplemental Materials

Targeted search in PubMed:

The search included the following terms and combinations: phthalates, ortho-phthalates, benzyl butyl phthalate, BBP, CAS 85-68-7, dibutyl phthalate, DBP, CAS 84-74-2, diisobutyl phthalate, DIBP, CAS 84-69-5, bis(2-ethylhexyl) phthalate, diethylhexyl phthalate, DEHP, CAS 117-81-7, dicyclohexyl phthalate, DCHP, CAS 84-61-7, human exposure, human studies, epidemiology studies, biomonitoring, serum, urine, placenta, cord blood, metabolite, children, pregnant women, women, men. The search was limited to 2003-2019.

Criteria for study inclusion and data extraction:

Although this was not a systematic review, we controlled for quality (e.g., whether covariates and confounders were included); however, we did not evaluate the studies for potential conflicts of interest or bias. We defined the following criteria:

1- Any of the five phthalates, BBP, DBP, DIBP, DEHP and DCHP or their metabolites was quantified (i.e., above the limit of detection used in the study) in any human tissue or bodily fluid.
2- Clear description of the population where the phthalates were measured (age, gender, life stage).
3- Clear description of the population where the endpoints were measured.
4- Statistically significant association between a concentration of phthalate and an endpoint measured.

Additionally, we reached out to study authors to request additional data or clarification on their results to ensure we included as many studies as there were available.

Of 64 studies, 38 met the criteria and were used in this case study. Of the 26 studies that were not included, there were:

1- Eight where the association between concentration and endpoint was either not statistically significant (e.g. > 0.05), or it was unclear what endpoints were significant or if the association found was significant;
2- Six studies where we could not identify the concentration(s) of the phthalates or metabolites measured that was associated with an endpoint although the study reported statistically significant correlation with an endpoint. For example, the data were presented as tertiles or quartiles without information on the range of concentration defining each of them; the data was presented a mean concentration but the endpoints were presented as multiple regressions;
3- Nine studies where the phthalates concentrations were expressed as low molecular weight (LMW) phthalates without specifying what chemicals were included in the group; phthalates were grouped as non-DEHP without specification;
4- One study did not have original data although it was not labeled as a review;
5- One study was a prospective study; and

6- One study where the metabolite that was statistically significant was from dioctyl phthalate, a compound that was not part of this case study.

Assumptions used in the intake estimation:

We used the following formula to estimate phthalates intake from urinary concentration of their metabolites:

Intake (µg/kg bw/d) = Metabolite concentration (µg/L) x (Vol (L)/day) x (1/bw (kg)) x (1/% elimination)

In cases when creatinine correction was needed, concentration of urinary metabolite in µg/g creatinine is multiplied by the urinary concentration of creatinine in g/L.

Metabolite concentration: The metabolites concentrations were reported using a variety of units including micromolar, microgram per liter of urine, micromole per gram of creatinine, microgram per gram of creatinine. The intake estimation formula uses µg/L, therefore all other units were converted to µg/L. When the concentration was given as molar sum of metabolites of DEHP, the concentration was standardized to the molecular weight of mono-2-ethyl-5-carboxypentyl phthalate (MECPP) (308 g/mol) (ng/mL) as recommended by Romano et al. (2018).

Creatinine: We used the mean urinary creatinine concentrations per persons 6-90 years of age NHANES 1988-1994 as reported by Barr et al. (2004). Our data set included ages ranging from children as young as 4 years of age to adults. For children, we used the mean concentration for 6-11 years-old (102.1 mg/dL) and for 12-19 years-old (161.5 mg/dL) from the NHANES data that best represented the age ranges included in our study. For the adult populations we used the following mean values: adult male: 157.5; adult female: 118.8 mg/dL. For pregnant women, we used the same value as for adult females.

Urine daily volume: We used 1.5 L and 1L for adults and children, respectively.

Body weight: We followed US Environmental Protection Agency 2011 Exposure Factors Handbook Table 8-1. The mean weights were as follows: 6-11 years: 32kg, 11-16 years: 57 kg, adult male and female: 80 kg. For pregnant women, we used the values listed on Table 8-29 Estimated body weight of pregnant women (NHANES 1999-2006); these are first trimester: 76 kg; second trimester: 73 kg; third trimester: 80 kg.

Fractional excretion of phthalates metabolites: We followed the European Chemicals Agency assumption that metabolites of BBP, DBP and DIBP are 100% excreted as monoesters. For DEHP, we used the mean percentage molar elimination estimated by Anderson et al. (2011). Mean percent elimination for MEHP was 6%, 5oxo MEHP 11%, 5OH MEHP 15% and 5cx MEHP 14%. When the data was presented as sum of DEHP metabolites, we used 50% molar elimination.

Examples of intake calculations:

DBP Intake (µg/kg bw/d) = MBP concentration (µg/L) x (Vol (L)/day) x (1/bw (kg)) x (1/% elimination)

DBP Intake in adult woman = 13.26 µg/g CRE x (0.1186 g CRE/dL x 100 dL/L) x 1.5L/d x (1/80 kg) x (1/100)

DBP Intake (µg/kg bw/d) = 0.029

DEHP Intake (µg/kg bw/d) = MEHP concentration (µg/L) x (Vol (L)/day) x (1/bw (kg)) x (1/% elimination)

DEHP Intake in children = 3.48 µg/L x 1L/d x (1/32 kg) x (1/6)

DEHP Intake (µg/kg bw/d) = 0.018

Table S1: List of publications that did not meet the criteria and were not included in the analysis

**Study Authors**	**Study title**	**Rationale for no inclusion**
Kold Jensen T et al.	Prenatal Exposure to Phthalates and Anogenital Distance in Male Infants from a Low-Exposed Danish Cohort (2010–2012). Environ Health Perspect 124:1107–1113; http://dx.doi.org/10.1289/ehp.1509870	No statistically significant associations were found.
Bornehag C-G et al.	Prenatal Phthalate Exposures and Anogenital Distance in Swedish Boys. Environ Health Perspect 123:101–107. http://dx.doi.org/10.1289/ehp.1408163	No statistically significant associations were found.
Jonsson AG et al.	Urinary Phthalate Metabolites and Biomarkers of Reproductive Function in Young Men. Epidemiology 2005;16: 487–493	No statistically significant associations were found.
Duty SM et al.	The Relationship Between Environmental Exposure to Phthalates and Computer-Aided Sperm Analysis Motion Parameters. Journal of Andrology 25:293, 2004	No statistically significant associations were found.
Hyland C et al.	Prenatal Exposure to Phthalates and Neurodevelopment in the CHAMACOS Cohort. Environmental Health Perspectives 127(10), 2019. https://doi.org/10.1289/EHP5165	No statistically significant associations were found.
Braun JM et al.	Gestational Exposure to Endocrine-Disrupting Chemicals and Reciprocal Social, Repetitive, and Stereotypic Behaviors in 4- and 5-Year-Old Children: The HOME Study. Environ Health Perspect 122:513–520. 2014 http://dx.doi.org/10.1289/ehp.1307261	No statistically significant associations were found.
James-Todd T et al.	The association between phthalates and metabolic syndrome: the National Health and Nutrition Examination Survey 2001–2010. Environmental Health (2016) 15:52. DOI 10.1186/s12940-016-0136-x	Unclear whether the association found was statistically significant.
Kobrosly RW et al.	Prenatal Phthalate Exposures and Neurobehavioral Development Scores in Boys and Girls at 6–10 Years of Age. Environ Health Perspect 122:521–528. http://dx.doi.org/10.1289/ehp.1307063	Unclear what data points were statistically significant.

Stahlhut RW et al.	Concentrations of Urinary Phthalate Metabolites Are Associated with Increased Waist Circumference and Insulin Resistance in Adult U.S. Males. Environ Health Perspect 115:876–882 (2007). doi:10.1289/ehp.9882	Quintiles for each metabolite were not defined.
Thurston SW et al.	Phthalate exposure and semen quality in fertile US men. Andrology. 2016 July ; 4(4): 632–638. doi:10.1111/andr.12124.	Unclear which metabolites concentrations were used.
Whyatt RM et al.	Prenatal Di(2-ethylhexyl)Phthalate Exposure and Length of Gestation Among an Inner-City Cohort. Pediatrics. 2009 December ; 124(6): e1213–e1220. doi:10.1542/peds.2009-0325	Unclear which concentrations were used in each model, expressed as quartile.
Wolff MS et al.	Associations of urinary phthalate and phenol biomarkers with menarche in a multiethnic cohort of young girls.	Significant correlation with a metabolite for dioctyl phthalate not included in this case study.
Arbuckle TE et al.	Processed data for CHMS 2007–2009: Bisphenol A, phthalates and lead and learning and behavioral problems in Canadian children 6–19 years of age. Data in Brief 8 (2016) 784–802. http://dx.doi.org/10.1016/j.neuro.2016.03.014	Couldn't identify urinary concentrations of phthalates metabolites.
Yolton K et al.	Prenatal Exposure to Bisphenol A and Phthalates and Infant Neurobehavior. Neurotoxicol Teratol. 2011 September ; 33(5): 558–566. doi:10.1016/j.ntt.2011.08.003	Couldn't identify concentrations significantly associated with endpoint .
Lien YJ et al.	Prenatal Exposure to Phthalate Esters and Behavioral Syndromes in Children at 8 Years of Age: Taiwan Maternal and Infant Cohort Study. Environ Health Perspect 123:95–100. http://dx.doi.org/10.1289/ehp.1307154	Couldn't identify urinary concentrations of phthalates metabolites.
Hatch EE et al.	Association of Endocrine Disruptors and Obesity: Perspectives from Epidemiologic Studies. Int J Androl. 2010 April ; 33(2): 324–332. doi:10.1111/j.1365-2605.2009.01035.x.	No original data.
Maresca MM et al.	Prenatal Exposure to Phthalates and Childhood Body Size in an Urban Cohort. Environ Health Perspect 124:514–520. http://dx.doi.org/10.1289/ehp.1408750	Data were expressed as non-DEHP rather than individual metabolites; it also included phthalates not covered in this study.
Katarina A et al.	Exposure to Bisphenols and Phthalates and Association with Oxidant Stress, Insulin Resistance, and Endothelial Dysfunction in Children. Pediatr Res. 2017 June ; 81(6): 857–864. doi:10.1038/pr.2017.16	Data expressed as low and high molecular weight phthalates rather than as individual concentrations. The groups also included more phthalates.
Philips EM et al.	Bisphenol and phthalate concentrations and its determinants among pregnant women in a	Data were expressed as high molecular weight phthalates.

	population-based cohort in the Netherlands, 2004–5. Environ Res. 2018; 161: 562–572. doi:10.1016/j.envres.2017.11.051	
Engel SM et al.	Prenatal Phthalate Exposure Is Associated with Childhood Behavior and Executive Functioning. Environ Health Perspect 118:565–571 (2010). doi:10.1289/ehp.0901470	Data expressed as low and high molecular weight phthalates without identification of what phthalates were included in each group.
Engel SM et al.	Prenatal Phthalate Exposure and Performance on the Neonatal Behavioral Assessment Scale in a Multiethnic Birth Cohort. Neurotoxicology. 2009 July ; 30(4): 522–528. doi:10.1016/j.neuro.2009.04.001	Data expressed as low and high molecular weight phthalates without identification of what phthalates were included in each group.
James-Todd T et al.	Urinary Phthalate Metabolite Concentrations and Diabetes among Women in the National Health and Nutrition Examination Survey (NHANES) 2001–2008. Environ Health Perspect 120:1307–1313 (2012). http://dx.doi.org/10.1289/ehp.1104717	Data expressed as quartile. No metabolites concentrations were included.
Wolff MS et al.	Prenatal Phenol and Phthalate Exposures and Birth Outcomes. Environ Health Perspect 116:1092–1097 (2008). doi:10.1289/ehp.11007	Data expressed as low and high molecular weight phthalates.
Wolff MS et al.	Phthalate exposure and pubertal development in a longitudinal study of US girls. Human Reproduction, Vol.29, No.7 pp. 1558–1566, 2014. doi:10.1093/humrep/deu081	Data expressed as low and high molecular weight phthalates.
Wolff MS et al.	Investigation of Relationships between Urinary Biomarkers of Phytoestrogens, Phthalates, and Phenols and Pubertal Stages in Girls. Environ Health Perspect 118:1039–1046 (2010). doi:10.1289/ehp.0901690	Data expressed as low and high molecular weight phthalates.
Song Y et al.	Urinary concentrations of bisphenol A and phthalate metabolites and weight change: a prospective investigation in US women. Int J Obes (Lond). 2014 December ; 38(12): 1532–1537. doi:10.1038/ijo.2014.63	Prospective study.
Kay VR et al.	Reproductive and developmental effects of phthalate diesters in females. Crit Rev Toxicol, 2013; 43(3): 200–219. DOI: 10.3109/10408444.2013.766149	Review.
Kim SH and Park MJ	Phthalate exposure and childhood obesity. Ann Pediatr Endocrinol Metab 2014;19:69-75. http://dx.doi.org/10.6065/apem.2014.19.2.69	Review.
Hannon and Flaws	The effects of phthalates on the ovary. Frontiers in Endocrinology 6:8, 2015. doi: 10.3389/fendo.2015.00008	Review.

Lovekamp T and Davis BJ	Mechanisms of Phthalate Ester Toxicity in the Female Reproductive System. Environ Health Perspect 111:139–145 (2003). doi:10.1289/ehp.5658	Review.

Supplemental Table S2: Significant neurological system's endpoints associated with estimated intakes lower than the reference doses for each phthalate

Phthalate	Trimester	Endpoint statistically significant	Outcome	Estimated intake [a]	Reference
DEHP	Second	Masculine play behavior in boys	Decreased	0.38 – 0.88	Swan et al. 2010
	Second	Odds of attention deficit hyperactivity disorder	Increased	2.66	Engel et al. 2018
	Third	Scores for delinquent, externalizing behavior	Positive	2.36	Huang et al. 2019
	Third	Psychomotor development in boys	Negative association	2.7	Kim et al. 2011
	Third	Scores for internalizing, externalizing problems	Higher scores	6.27	Huang et al. 2019
DBP	Second	Emotional symptom score, internalizing behavior, relationship problems	Increased	0.68	Philippat et al. 2017
	Third	Mental and psychomotor development in boys	Negative association	0.38 – 0.71	Kim et al. 2011; Whyatt et al. 2012
	Third	Odds of motor delay and clinically withdrawn behavior	Increased	0.71	Whyatt et al. 2012
	Third	Full scale IQ, perceptual reasoning, processing speed, and working memory	Negative association	1.50	Factor-Litvak et al. 2014
BBP	Second	Internalizing behavior		0.26	Philippat et al. 2017
	Third	Odds of clinically withdrawn and internalizing behavior	Increased	0.35	Whyatt et al. 2012
	Third	Social problems	Higher scores	0.07 – 0.23	Huang et al. 2019
	Third	Perceptual reasoning	Negative association		Factor-Litvak et al. 2014
DIBP	Second	Masculine play behavior in boys	Decreased	0.08	Swan et al. 2010
	Third	Odds of motor delay	Increased	0.17	Whyatt et al. 2012
	Third	Psychomotor development	Decreased	0.17	Whyatt et al. 2012
	Third	Full scale IQ, perceptual reasoning, processing speed, and working memory	Negative association	0.35	Factor-Litvak et al. 2014

[a] In microgram per kilogram of body weight per day
Abbreviations: DEHP: diethylhexyl phthalate; DBP: dibutyl phthalate; BBP: butylbenzyl phthalate; DIBP: diisobutyl phthalate; IQ: intelligence quotient; μg/kg-bw.d: microgram per kilogram of body weight per day

Supplemental Table S3: Significant metabolic systems' endpoints associated with estimated intakes lower than the reference doses for each phthalate

Phthalate	Population	Endpoint statistically significant	Outcome	Estimated intake [a]	Reference
	Thyroid				
DEHP	Boys and girls (12-19 years)	Thyroid hormone T3	Decreased	2.2 – 2.89	Meeker et al. 2011
	Women	Free thyroid hormone T4	Negative association	0.53 – 1.79	Johns et al. 2015
	Men	Free thyroid hormone T4 and T3	Negative association	0.99	Meeker et al. 2007
	Men	Thyroid stimulating hormone	Decreased	242.55	Wang et al. 2018
DBP	Children (4-9 years)	Thyroid hormone T3	Decreased	1.97 – 2.34	Boas et al. 2010
BBP	Children (4-9 years)	Thyroid hormone T3	Decreased	0.10	Boas et al. 2010
	Pancreas				
DEHP	Children (12 years)	Insulin sensitivity index in obese pre-pubertal	Positive correlation	0.19	Smerieri et al. 2015
	Boys and girls (12-19 years)	Homeostatic Model Assessment of Insulin Resistance (HOMA-IR)	Positive correlation	2.76	Trasande et al. 2013
	Men and women	Homeostatic Model Assessment of Insulin Resistance (HOMA-IR)	Positive correlation	1.56 - 3.16	Meeker et al. 2011; Kim et al. 2013
DIBP	Women	Glucose levels	Increased	0.20	James-Todd et al. 2018
	Fat tissue				
DEHP	Girls (4-7 years). Phthalates were measured in the mother (3T)	Body mass index z-score in girls	Negative correlation	1.77	Buckley et al. 2016

	Children (8-10 years)	Obesity in girls	Negative association	1.71- 4.31	Zang et al. 2014
	Women	Body mass index	Positive association	0.56	Yaghiyan et al. 2015
	Men and women	Body mass index and waist circumference	Negative association	13.75	Hatch et al. 2008
DBP	Children (8-10 years)	Obesity in boys	Positive association	0.95	Zang et al. 2014
	Women	Body mass index and waist circumference	Positive association	0.30	Hatch et al. 2008
	Men and women	Body mass index	Positive association	0.72	Hatch et al. 2008
BBP	Men and women	Body mass index and waist circumference	Positive association	0.58	Hatch et al. 2008

[a] In microgram per kilogram of body weight per day
Abbreviations: DEHP: diethylhexyl phthalate; DBP: dibutyl phthalate; BBP: butylbenzyl phthalate; DIBP: diisobutyl phthalate; T: trimester; µg/kg-bw.d: microgram per kilogram of body weight per day

Supplemental Table S4: Significant reproductive system endpoints associated with estimated intakes lower than the reference doses for each phthalate

Phthalate	Population	Endpoint statistically significant	Outcome	Estimated intake [a]	Reference
DEHP	Boys. Phthalates were measured in the mother (1T)	Anogenital distance	Negative correlation	0.66 – 0.80	Swan et al. 2015
	Girls (8 years)	Puberty	Delayed	4.63	Kasper-Sonnenberg et al. 2017
	Women	Ovarian antral follicle count	Decreased	0.03 – 0.25	Messerlian et al. 2016
	Women	Fertilized eggs, mature oocytes, top quality oocytes, total oocytes	Decreased	0.23 – 1.39	Machtinger et al. 2018
	Women	Gestational length and odds of C-section	Increased	2.56 – 4.20	Adibi et al. 2009
	Women	Gestational age (premature labor)	Shorter	2.46	Boss et al. 2018
	Women	Trophoblast differentiation genes	Negative association	4.21 – 4.45	Adibi et al. 2010
	Men	Semen quality	Decreased	242.55	Wang et al. 2018
DBP	Girls (8 years)	Puberty	Delayed	1.56	Kasper-Sonnenberg et al. 2017
	Women	Ovarian antral follicle count	Decreased	0.24 – 0.41	Messerlian et al. 2016
	Women	Fertilized eggs, mature oocytes, total oocytes	Decreased	0.71	Machtinger et al. 2018
	Men	Decreased sperm motility and concentration	Decreased	0.20 – 0.38	Hauser et al. 2006; Duty et al. 2003
BBP	Girls (8 years)	Puberty		0.19	Kasper-Sonnenberg et al. 2017
	Men	Sperm motility	Decreased	0.25	Duty et al. 2003
DIBP	Women	Ovarian antral follicle count	Decreased	0.13-0.19	Messerlian et al. 2016
	Women	Trophoblast differentiation genes	Negative association	0.27	Adibi et al. 2010

Reproductive hormones and associated globulins					
DEHP	Boys (8-14 years). Phthalates were measured in the mother (3T)	Free testosterone	Decreased	1.52	Ferguson et al. 2014
		Steroid hormone binding globulin	Increased	1.52 – 5.09	Ferguson et al. 2014
	Women	Serum inhibin	Decreased	2.04	Du et al. 2018
	Men	Free testosterone, total testosterone, estradiol, free androgen index	Decreased	0.41 – 2.12	Mendiola et al. 2012
	Men	Testosterone/estradiol ratio	Positive association	0.99	Meeker et al. 2009
	Men	Steroid hormone binding globulin	Increased	0.41	Mendiola et al. 2012
DBP	Boys (8-14 years). Phthalates were measured in the mother (3T)	Steroid hormone binding globulin	Increased	0.63	Ferguson et al. 2014
		Dehydroepiandrosterone sulfate	Decreased	0.63	Ferguson et al. 2014
BBP	Boys (8-14 years). Phthalates were measured in the mother (3T)	Steroid hormone binding globulin	Increased	0.06	Ferguson et al. 2014
DIBP	Boys (8-14 years). Phthalates were measured in the mother (3T)	Free testosterone, total testosterone, estradiol	Decreased	0.18	Ferguson et al. 2014

[a] In microgram per kilogram of body weight per day
Abbreviations: DEHP: diethylhexyl phthalate; DBP: dibutyl phthalate; BBP: butylbenzyl phthalate; DIBP: diisobutyl phthalate; T: trimester

References

Adibi JJ, Hauser R, Williams PL, Whyatt RM, Calafat AM, Nelson RH, et al. 2009. Maternal Urinary Metabolites of Di-(2-Ethylhexyl) Phthalate in Relation to the Timing of Labor in a US Multicenter Pregnancy Cohort Study. Am J Epidemiol 169:1015–1024, PMID 19251754 DOI: 10.1093/aje/kwp001

Adibi JJ, Whyatt RM, Hauser R, Bhat HK, Davis BJ, Calafat AM, et al. 2010. Transcriptional Biomarkers of Steroidogenesis and Trophoblast Differentiation in the Placenta in Relation to Prenatal Phthalate Exposure. Environ Health Perspect 118:291–296, PMID: 20123604 DOI: 10.1289/ehp.0900788

Anderson WAC, Castle L, Hird S, Jeffery J, Scotter MJ. 2011. A twenty-volunteer study using deuterium labelling to determine the kinetics and fractional excretion of primary and secondary urinary metabolites of di-2-ethylhexylphthalate and di-iso-nonylphthalate. Food Chem Toxicol 49:2022–2029, PMID: 21609750 DOI: 10.1016/j.fct.2011.05.013

Barr DB, Wilder LC, Caudill SP, Gonzalez AJ, Needham LL, Pirkle JL. 2005. Urinary Creatinine Concentrations in the U.S. Population: Implications for Urinary Biologic Monitoring Measurements. Environ Health Perspect 113:192-200, PMID: 15687057, DOI: 10.1289/ehp.7337

Boas M, Frederiksen H, Feldt-Rasmussen U, Skakkebak NE, Hegefus L, Hilsted L et al. 2010. Childhood Exposure to Phthalates: Associations with Thyroid Function, Insulin-like Growth Factor I, and Growth. Environ Health Perspect 118:1458–1464, PMID: 20621847, DOI: 10.1289/ehp.0901331

Boss J, Zhai J, Aung MT, Ferguison KK, Johns LE, McElrath TF, et al. 2018. Associations between mixtures of urinary phthalate metabolites with gestational age at delivery: a time to event analysis using summative phthalate risk scores. Environmental Health 17:56 PMID: 29925380, DOI: 10.1186/s12940-018-0400-3

Buckley JP, Engel SM, Braun JM, Whyatt RM, Daniels JL, Mendez MA, et al. 2016. Prenatal phthalate exposures and body mass index among 4 to 7 year old children: A pooled analysis. Epidemiology 27: 449–458, PMID: 26745610, DOI: 10.1097/EDE.0000000000000436

Du Y-Y, Guo N, Wang Y-X, Hua X, Deng T-R, Teng X-M, et al. 2018. Urinary phthalate metabolites in relation to serum anti-Müllerian hormone and inhibin B levels among women from a fertility center: a retrospective analysis. Reprod Health 15:33 PMID: 29471860, DOI: 10.1186/s12978-018-0469-8

Duty SM, Silva MJ, Barr DB, Brock JW, Ryan L, Chen Z, et al. 2003. Phthalate Exposure and Human Semen Parameters. Epidemiology 14:269 –277, PMID: 12859026

Engel SM, Villanger GD, Nethery RC, Thomsen C, Saki AK, Drover SSM, et al. 2018. Prenatal Phthalates, Maternal Thyroid Function, and Risk of Attention-Deficit Hyperactivity Disorder in the Norwegian Mother and Child Cohort. Environ Health Perspect 126:057004, PMID: 29790729, DOI: 10.1289/EHP2358

Factor-Litvak P, Insel B, Calafat AM, Liu X, Perera F, Rauh VA, et al. 2014. Persistent Associations between Maternal Prenatal Exposure to Phthalates on Child IQ at Age 7 Years. PLOS One 9(12):e114003, PMID: 25493564, DOI: 10.1371/journal.pone.0114003

Ferguson KK, Peterson KE, Lee JM, Mercado-Garcia A, Blank-Goldenberg C, Tellez-Rojo MM, et al. 2014. Prenatal and peripubertal phthalates and bisphenol A in relation to sex hormones and puberty in boys. Reprod Toxicol 47:70–76, PMID: 24945889, DOI: 10.1016/j.reprotox.2014.06.002

Hatch EE, Nelson JW, Mustafa Qureshi M, Weinberg J, Moore LL, Singer M, et al. 2008. Association of urinary phthalate metabolite concentrations with body mass index and waist circumference: a cross-sectional study of NHANES data, 1999–2002. Environ Health 7:27, PMID: 18522739, DOI: 10.1186/1476-069X-7-27

Hauser R, Meeker JD, Duty S, Silva MJ, Calafat AM. 2006. Altered Semen Quality in Relation to Urinary Concentrations of Phthalate Monoester and Oxidative Metabolites. Epidemiology 17: 682-691, PMID: 17003688, DOI: 10.1097/01.ede.0000235996.89953.d7

Huang H-B, Kuo P-H, Su P-H, Sun C-W, Chen WJ, Wang S-L. 2019. Prenatal and childhood exposure to phthalate diesters and neurobehavioral development in a 15-year follow-up birth cohort study. Environ Res 172:569–577, PMID: 30875510, DOI: 10.1016/j.envres.2019.02.029

Huang P-C, Kuo P-L, Guo Y-L, Liao P-C, Lee C-C. 2007. Associations between urinary phthalate monoesters and thyroid hormones in pregnant women. Hum Reprod 22:2715–2722, PMID: 17704099, DOI: 10.1093/humrep/dem205

Huang P-C, Tsai CH, Liang WY, Li SS, Huang HB, Kuo PL. 2016. Early Phthalates Exposure in Pregnant Women Is Associated with Alteration of Thyroid Hormones. PLoS ONE 11(7):e0159398, PMID: 27455052, DOI: 10.1371/journal.pone.0159398

James-Todd TM, Chiu Y-H, Messerlian C, Minguez-Alarcon L, Ford JB, Keller M, et al. 2018. Trimester-specific phthalate concentrations and glucose levels among women from a fertility clinic. Environ Health 17:55, PMID: 29898728, DOI: 10.1186/s12940-018-0399-5

Johns LE, Ferguson KK, Soldin OP, Cantonwine DE, Rivera-Gonzalez LO, Anzalota del Toro LV, et al. 2015. Urinary phthalate metabolites in relation to maternal serum thyroid and sex hormone levels during pregnancy: a longitudinal analysis. Reprod Biol Endocrinol 13:4, PMID: 25596636, DOI: 10.1186/1477-7827-13-4

Kasper-Sonnenberg M, Wittsiepe J, Wald K, Koch HM, Willhem M. 2017. Pre-pubertal exposure with phthalates and bisphenol A and pubertal development. PLoS ONE 12(11):e0187922, PMID: 29155850, DOI: 10.1371/journal.pone.0187922

Kim JH, Park HY, Bae S, Lim Y-H, Hong Y-C. 2013. Diethylhexyl Phthalates Is Associated with Insulin Resistance via Oxidative Stress in the Elderly: A Panel Study. PLoS ONE 8(8): e71392, PMID: 23977034, DOI: 10.1371/journal.pone.0071392

Kim Y, Ha E-H, Kim E-J, Park H, Ha M, Kim J-H et al. 2011. Prenatal Exposure to Phthalates and Infant Development at 6 Months: Prospective Mothers and Children’s Environmental Health (MOCEH) Study. Environ Health Perspect 119:1495–1500, PMID: 21737372, DOI: 10.1289/ehp.1003178

Kock HM, Bolt HM, Preuss R, Angerer J. 2005. New metabolites of di(2-ethylhexyl)phthalate (DEHP) in human urine and serum after single oral doses of deuterium-labelled DEHP. Arch Toxicol 79: 367-376, PMID: 15700144, DOI: 10.1007/s00204-004-0642-4

Machtinger RM, Hauser R, Liang L, Mansur A, Adir M, Dioni L, et al. 2018. Urinary concentrations of biomarkers of phthalates and phthalate alternatives and IVF outcomes. Environ Int 111: 23–31, PMID: 30481674, DOI: 10.1016/j.envint.2018.11.043

Meeker JD, Calafat AM, Hauser R. 2007. Di(2-ethylhexyl) Phthalate Metabolites May Alter Thyroid Hormone Levels in Men. Environ Health Perspect 115:1029-1034, PMID: 17637918, DOI: 10.1289/ehp.9852

Meeker JD, Calafat AM, Hauser R. 2009. Urinary metabolites of di(2-ethylhexyl) phthalate are associated with decreased steroid hormone levels in adult men. J Androl 30: 287-297, PMID: 19059903, DOI: 10.2164/jandrol.108.006403

Meeker JD, Ferguson KK. 2011. Relationship between Urinary Phthalate and Bisphenol A Concentrations and Serum Thyroid Measures in U.S. Adults and Adolescents from the National Health and Nutrition Examination Survey (NHANES) 2007–2008. Environ Health Perspect 119:1396-1402, PMID: 21749963, DOI: 10.1289/ehp.1103582

Mendiola J, Meeker JD, Jorgensen N, Andersson AM, Liu F, Calafat AM, et al. 2012. Urinary concentrations of di(2-ethylhexyl) phthalate metabolites and serum reproductive hormones: Pooled analysis of fertile and infertile men. Androl. 33: 488-498, PMID: 21597090, DOI: 10.2164/jandrol.111.013557

Messerlian C, Souter I, Gaskins AJ, Williams PK, Ford JB, Chiu Y-H, et al. 2016. Urinary phthalate metabolites and ovarian reserve among women seeking infertility care. Hum Reprod 31:75-83, PMID: 26573529, DOI: 10.1093/humrep/dev292

Philippat C, Nakiwala D, Calafat AM, Bottom J, De Agostini M, Heude B, et al. 2017. Prenatal Exposure to Nonpersistent Endocrine Disruptors and Behavior in Boys at 3 and 5 Years. Environ Health Perspect 125:097014, PMID: 28937960, DOI: 10.1289/EHP1314

Smerieri A, Testa C, Lazzeroni P, Nuti F, Grossi E, Cesari S, et al. 2015. Di-(2-Ethylhexyl) Phthalate Metabolites in Urine Show Age-Related Changes and Associations with Adiposity and Parameters of Insulin Sensitivity in Childhood. PLoS ONE 10(2): e0117831, PMID: 25706863, DOI: 10.1371/journal.pone.0117831

Soomro MH, BaizN, Philippat C, Vernet C, Siroux V, Maesano CN, et al. 2018. Prenatal Exposure to Phthalates and the Development of Eczema Phenotypes in Male Children: Results from the EDEN Mother–Child Cohort Study. Environ Health Perspect 126:027002, PMID: 29398652, DOI: 10.1289/EHP1829

Swan SH, Liu F, Hines M, Kruse RL, Wang C, Redmon JB, et al. 2010. Prenatal phthalate exposure and reduced masculine play in boys. Int J Androl 33:259-269, PMID: 19919614, DOI: 10.1111/j.1365-2605.2009.01019.x

Swan SH, Sathyanarayana S, Barret ES, Janssen S, Liu F, Nguyen RHN, et al. 2015. First trimester phthalate exposure and anogenital distance in newborns. Hum Reprod 30:963-972, PMID: 25697839, DOI: 10.1093/humrep/deu363

Trasande L, Sathyanarayana S, Spanier AJ, Trachtman H, Attina TM, Urbina EM. 2013a. Urinary Phthalates Are Associated with Higher Blood Pressure in Childhood. J Pediatr 163: 747-53, PMID: 23706605, DOI: 10.1016/j.jpeds.2013.03.072

Trasande L, Sathyanarayana S, Trachtman H. 2014. Dietary Phthalates and Low-Grade Albuminuria in US Children and Adolescents. Clin J Am Soc Nephrol 9: 100-109, PMID: 24178978, DOI: 10.2215/CJN.04570413

Trasande L, Spanier AJ, Sathyanarayana S, Attina T, Blustein J. 2013b. Urinary Phthalates and Increased Insulin Resistance in Adolescents. Pediatrics 132:e646–e655, PMID: 23958772, DOI: 10.1542/peds.2012-4022

US Environmental Protection Agency (EPA). 2011. Exposure Factors Handbook 2011 Edition (Final Report). https://cfpub.epa.gov/ncea/risk/recordisplay.cfm?deid=236252

Wang Y-X, Zhou B, Chen Y-J, Liu C, Huang L-L, Liao J-Q, et al. 2018. Thyroid function, phthalate exposure and semen quality: Exploring associations and mediation effects in reproductive-aged men. Environ Int 116:278-285, PMID: 29704806, DOI: 10.1016/j.envint.2018.04.031

Whyatt RM, Liu X, Rauh VA, Calafat AM, Just AC, Hoepner L, et al. 2012. Maternal Prenatal Urinary Phthalate Metabolite Concentrations and Child Mental, Psychomotor, and Behavioral Development at 3 Years of Age. Environ Health Perspect 120:290-295, PMID: 21893441, DOI: 10.1289/ehp.1103705

Yaghiyan L, Sites S, Ruan Y, Chang S-H. 2015. Associations of Urinary Phthalates with Body Mass Index, Waist Circumference, and Serum Lipids Among Females: National Health and Nutrition Examination Survey 1999–2004. Int J Obes (Lond) 39: 994-1000, PMID: 25644057, DOI: 10.1038/ijo.2015.8

Zhang Y, Meng X, Chen K, Li D, Zhao K, Zhao Y, et al. 2014. Age and Sex-Specific Relationships between Phthalate Exposures and Obesity in Chinese Children at Puberty. PLoS ONE 9(8): e104852, PMID: 25121758, DOI: 10.1371/journal.pone.0104852
